# Mobile Phone–based Infectious Disease Surveillance System, Sri Lanka

**DOI:** 10.3201/eid1610.100249

**Published:** 2010-10

**Authors:** Colin Robertson, Kate Sawford, Samson L.A. Daniel, Trisalyn A. Nelson, Craig Stephen

**Affiliations:** Author affiliations: University of Victoria, Victoria, British Columbia, Canada (C. Robertson, T.A. Nelson);; Wilfrid Laurier University, Waterloo, Ontario, Canada (C. Robertson);; University of Calgary, Calgary, Alberta, Canada (K. Sawford, C. Stephen);; Ministry of Livestock Development, Colombo, Sri Lanka (S.L.A. Daniel)

**Keywords:** Disease surveillance, early warning, zoonoses, animal health, low and middle income countries, resource-limited settings, Sri Lanka, sentinels, research

## Abstract

Because many infectious diseases are emerging in animals in low-income and middle-income countries, surveillance of animal health in these areas may be needed for forecasting disease risks to humans. We present an overview of a mobile phone–based frontline surveillance system developed and implemented in Sri Lanka. Field veterinarians reported animal health information by using mobile phones. Submissions increased steadily over 9 months, with ≈4,000 interactions between field veterinarians and reports on the animal population received by the system. Development of human resources and increased communication between local stakeholders (groups and persons whose actions are affected by emerging infectious diseases and animal health) were instrumental for successful implementation. The primary lesson learned was that mobile phone–based surveillance of animal populations is acceptable and feasible in lower-resource settings. However, any system implementation plan must consider the time needed to garner support for novel surveillance methods among users and stakeholders.

Emerging infectious diseases in animals and humans are being identified more frequently, many in low-income tropical countries, and this trend is expected to continue (*1*). Because ≈75% of these diseases in humans have originated in animals (*1*), interest has increased considerably in the utility of animal health surveillance for prediction of human health risks (*2**–**5*). The Canary Database, an online database named after the canary in the coal mine analogy, demonstrates the broad interest in this idea; it contains >1,600 articles related to animal sentinels of zoonotic, environmental, and toxic effects on human health (*6*). However, in practice, establishing links between animal and human health data has been difficult because data from animal and human health surveillance systems are obtained at different resolutions and scales and for different purposes. Human health surveillance is often based on aggregated diagnoses data obtained from standardized electronic medical records. Animal health surveillance systems vary widely (*7*). Where electronic veterinary records are kept, data can be extracted to central databases and analyzed. However, in lower-resource settings, electronic recording of veterinary services is often not feasible.

In many human health projects in resource-challenged areas, mobile technologies have emerged as a promising solution for obtaining, transmitting, and analyzing human health information in a timely fashion (*8**–**11*). In Peru, a mobile phone–based surveillance system has been used for early detection of infectious disease outbreaks in the Peruvian Navy (*12*). In Africa, the Satellife project has been using mobile data collection devices for >2 decades in human health surveys, and a project is under way that uses mobile phones and wireless technology for disease surveillance in Uganda (*13*). Many United Nations health and development projects in Africa now use mobile phones for obtaining field data (*14*). However, we are not aware of any examples of mobile phone–based disease surveillance that supports an animal-based emerging infectious disease system in the developing world.

In response to these challenges, we have developed the Infectious Disease Surveillance and Analysis System (IDSAS), a mobile phone–based surveillance system specific for animal populations in lower-resource settings. A pilot version of this system was implemented in January 2009 in partnership with the Department of Animal Production and Health (DAPH) in Sri Lanka. The objective of this system is to obtain animal health information from field veterinarians in a timely fashion to establish baseline patterns in animal health. By establishing these baseline patterns through regular electronic surveillance, we aim to build capacity to detect changes that may facilitate early detection of changing risks for emerging infectious diseases. We describe the design and implementation of the system, present preliminary data on submission patterns, provide examples of data that are being obtained, and discuss obstacles and opportunities encountered during the first 9 months of operation. This report highlights and generalizes some of the lessons learned during the planning and implementation of the IDSAS in Sri Lanka.

## Materials and Methods

### Veterinary Services in Sri Lanka

Veterinary services in Sri Lanka are provided largely by the DAPH, a national-level body responsible for control of livestock diseases, livestock research, animal breeding, and education in animal husbandry. Veterinary services are delivered through provincial-level DAPH councils and field offices. Provinces are made up of districts, which are further divided into divisional secretariat. Each divisional secretariat is assigned a field veterinarian who is responsible for providing animal health services within that division.

### System Structure

**Figure 1:**
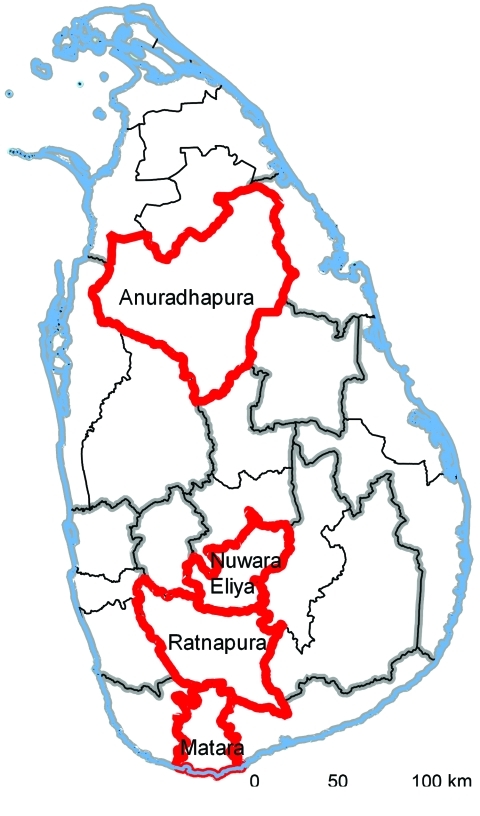
Study districts in Sri Lanka where field veterinarians participated in the Infectious Disease Surveillance and Analysis System and obtained data on animal health during their daily work activities. Study districts are indicated by red outlines; provincial boundaries are indicated in gray, and district boundaries are indicated in black.

Forty field veterinarians were recruited to pilot the IDSAS in 4 districts in separate provinces. The districts (Nuwara Eliya, Anuradhapura, Matara, Ratnapura) were selected to capture variation in livestock practices, climate, and environment (Figure 1).

Capacity for electronic collection and submission of data was developed in the IDSAS to decrease the time from detecting to reporting animal health events from that of the existing method of mailed written reports. Internet access is limited in many parts of Sri Lanka, but the cellular phone network is extensive. Mobile phones (Palm-Centro Smartphones; Palm, Inc., Sunnyvale, CA, USA) were used as the data collection platform. Animal health surveys were developed by using EpiSurveyor, a free and open-source software package developed for obtaining public health data (www.datadyne.org). EpiSurveyor has been used extensively for human health data collection in Africa.

**Figure 2:**
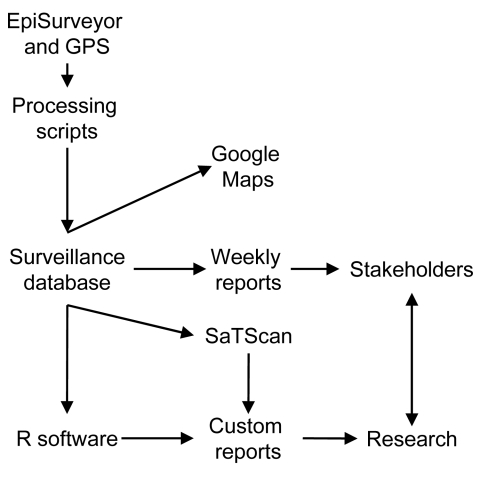
Schematic overview of major components of the Infectious Disease Surveillance and Analysis System, Sri Lanka. GPS, global positioning system; stakeholders, groups and persons (field veterinarians, administrators, and researchers) whose decision-making and actions are affected by emerging infectious diseases and animal health.

Surveys were filled out in remote areas without cellular service and transmitted when the user returned to an area of reception. Decoupling data collection from transmission-capable locations greatly expanded the geographic range of the surveillance system. The location of each survey was also collected with global positioning system (GPS) software and an external receiver connected to the phone by Bluetooth (www.bluetooth.com/English/Pages/default.aspx). Field veterinarians obtained data from their daily working activities (clinic and farm visits). Survey and GPS data were encoded and transmitted to a central database by email at the end of each day. A schematic overview of the IDSAS is shown in Figure 2.

### Information Structure

The pilot study was restricted to health issues in chickens, cattle, and buffalo. Every time a field veterinarian visited a farm or saw a case involving 1 of these 3 species, a survey was completed by using EpiSurveyor, and the location (for farm visits) was recorded. Although we sought daily submissions, our minimum target submission rate was 2 surveys/field veterinarian/wk. This value was based on an estimate of the number of cases in chickens, cattle, and buffalo seen on average by field veterinarians and on work-related disruptions that could interfere with data submission (training, sick days, holidays).

The first draft of the survey was based on the Alberta Veterinary Surveillance Network Veterinary Practice Surveillance initiative (*15*). In the second stage, the survey was reviewed with several field veterinarians and government employees within the DAPH to ensure that it was applicable to veterinary practice in Sri Lanka. Most questions were single-answer, multiple-choice questions, although additional comments were allowed in a free-text field. The survey was designed to minimize the time required to fill out each survey, reduce the number of data entry errors, and enable easier and automated data analysis.

Data for each case included date, location, type of operation, nature of visit (routine/nonroutine), age and sex of affected animal, number on farm, number affected, clinical syndrome, clinical diagnosis, laboratory testing if applicable, and other animals on the premises. A survey could contain up to 3 cases if all 3 species were present on a farm. Field veterinarians selected from clinical syndromes shown in Table 1. Within EpiSurveyor, each syndromic grouping was linked to a list of clinical diagnoses.

**Table 1:** Syndrome groupings in animal health surveys in the Infectious Disease Surveillance and Analysis System, Sri Lanka, January 1–September 30, 2009

Species	Syndrome grouping
Buffalo and cattle	Abortion/birth defect
	Ambulatory lameness
	Decreased feed intake/milk production
	Gastrointestinal signs
	Neurologic signs
	Recumbency
	Peripheral edema/miscellaneous swelling
	Reproduction/obstetrics problems
	Respiratory
	Skin/ocular/mammary
	Sudden or unexplained death
	Urologic
	Vesicular/ulcerative
	Other
Chickens	Ambulatory
	Decreased egg production, weight gain, and appetite
	Neurologic/recumbent
	Peripheral edema/miscellaneous swelling
	Respiratory
	Skin/ocular
	Sudden or unexplained death
	Other

### Reporting and Data Analysis

Data reported represent the experience of the IDSAS during January 1–September 30, 2009. Weekly surveillance reports were disseminated to project partners containing a list of cases. These reports documented the following details pertaining to each case submitted during the previous week: date, species, reported syndrome, suspected clinical diagnosis, number of animals affected, number of animals on farm, number of dead animals, and a flag indicating whether samples were submitted to a laboratory.

#### Data Completeness and Submission Patterns

Measures of data completeness used for the IDSAS at the planning and early implementation stages followed the guidelines of Lescano et al. (*16*). In the planning stage, assessment of the workload of data collectors is essential to determine whether data can be obtained with existing resources. The IDSAS data collection procedure involved separate software programs for animal health surveys and GPS data collection. These data were linked by a common identifier entered by field veterinarians at the time of survey completion. To explore the link between survey and GPS data, we report completeness for surveys, GPS points, and linked survey–GPS records. We also report the percentage of surveys with a linked GPS point. Because field veterinarians worked 6 days per week, we expected a day-of-the-week effect and therefore examined variation in survey submission by day of the week. Finally, we examined weekly submission counts to determine temporal patterns and fitted a linear trend model to weekly counts to determine the average change in submissions per week.

#### Statistical Surveillance

Digital storage of data that otherwise might not be captured enables more sophisticated statistical analysis. To demonstrate how the IDSAS database could be used in an outbreak detection context, we present an example of statistical surveillance by using the total number of weekly surveys submitted by participating field veterinarians as an indicator for unusual animal health events. We used these data in a prospective temporal surveillance cumulative sum (CUSUM) statistic implemented in the statistical software package R (*17*). The CUSUM measures accumulations of extra variance in a sequential framework, and alarms are signaled when the statistic exceeds a specified threshold. Parameters are required for the expected value, the reference value *k*, and the alarm threshold *h*. We estimated values for *k* and *h* on the basis of an expected false-positive rate of 1 every 52 weeks to detect a change that was 2 SD above the reference value. We evaluated 2 baseline scenarios: the mean of the first 14-week period, and a set value of 100 surveys per week. Data were analyzed weekly beginning at week 14 until the end of the study.

#### Caseload and Case Profile

The distribution of cases is presented as divided by species and district. We also present the frequency of the 5 most commonly reported syndromes for each species.

#### Assessing System Implementation

The experience of implementing the IDSAS provides lessons for future surveillance projects in lower-resource settings. We synthesize some of the key lessons learned during this phase of the IDSAS on the basis of technical, financial, political, and ethical/societal/cultural considerations (*18*).

## Results

### Data Completeness and Submission Patterns

The IDSAS operated for 273 days. During this period, 3,981 unique surveys were submitted to the system by participating field veterinarians. This value corresponds to ≈99 surveys/field veterinarian over a 9-month period (11 per month); our intended submission target was a minimum of 2 submissions/field veterinarian/week. During this period, 96% of days had >1 conducted survey. A total of 1,650 unique GPS points were submitted, of which 1,172 (71%) were linked to an associated survey. For the total days under surveillance, GPS data were collected for 76%, and GPS and survey data were recorded for 64%. Informal discussions with many field veterinarians showed that it took ≈1 minute to complete an animal health survey and 1 minute to obtain a GPS point once the IDSAS had been in place for 6 months.

**Figure 3:**
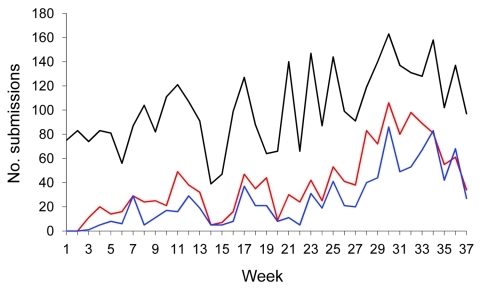
Number of survey (black line), global positioning system (red line), and linked survey–global positioning system (blue line) submissions to the Infectious Disease Surveillance and Analysis System, by week, Sri Lanka, January 1–September 30, 2009.

A generally increasing overall trend in temporal patterns in submissions (Figure 3) was evident. The linear trend model showed a significant weekly increase in submissions of 1.65% (p<0.001, R^2^ = 0.31). The trend was also characterized by large variation (coefficient of variation 3.01), with a large decrease (39 surveys) in submissions during week 14. As expected, day-of-the-week variation was present in submissions, and weekly survey counts were 306 on Saturdays and 326 on Sundays. During the week, totals ranged from 515 to 695.

### Statistical Surveillance

On the basis of the parameters described, the reference value *k* was ≈2.6 and the threshold value *h* was ≈4.1. Using week 14 as a baseline, we expected 84 weekly visits, which in the CUSUM analysis flagged an alarm at week 26 and weeks 30 through the end of the study (week 38). Using the expected value of 100 weekly visits, we determined that alarms were signaled during weeks 31–38.

### Caseload and Case Profile

**Figure 4:**
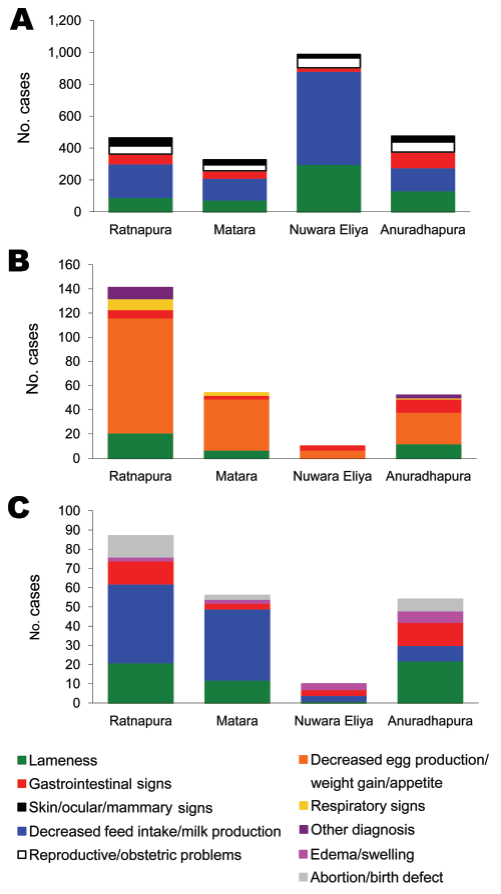
Frequency of syndrome groups seen by field veterinarians in cattle (A), buffalo (B), and chickens (C) in 4 study districts as part of the Infectious Disease Surveillance and Analysis System, Sri Lanka, January 1–September 30, 2009.

Of 3,981 surveys submitted during the 9 months of operation, 3,150 cases were reported (i.e., reported an animal health issue). Most (83%) cases were in cattle, followed by chickens and buffaloes (Table 2). These cases were mostly from an area known to contain a large number of dairy cattle operations. Production-related syndromes were the most commonly reported across all species, with decreased feed intake/milk production most prevalent in cattle and buffaloes, and decreased egg production/weight gain/appetite in chickens (Figure 4). In buffaloes, markedly higher gastrointestinal and lameness submissions were noted relative to other syndrome groupings. Gastrointestinal signs were common in Anuradhapura across all species. Cases in chickens were found predominantly in Ratnapura, where there are a large number of poultry operations. Syndrome profiles for chickens were similar across all districts (Figure 4).

**Table 2:** Cases in animals in the 4 study districts covered by the Infectious Disease Surveillance and Analysis System, Sri Lanka, January 1–September 30, 2009*

District	No. cattle cases	No. buffalo cases	No. chicken cases	Total no. cases
Ratnapura	548	106	146	800
Matara	388	62	55	505
Nuwara Eliya	1,095	16	11	1,122
Anuradhapura	596	70	57	723
Total	2,627	254	269	3,150

*Cases are defined as animal health issues.

### Alerts Identified by the IDSAS

In 1 instance, suspected cases of black quarter (*Clostridium chauvoei* bacterial infection) were identified at the time of review of the weekly report review. Because the DAPH was made aware of the cases shortly after they were identified by the field veterinarian, it was able to confirm that the field veterinarian obtained tissue samples for diagnostic testing. This increased information flow would not have been possible in the DAPH surveillance program because written reports of suspected cases from field veterinarians are received monthly, and each must be reviewed individually to identify suspected cases of a particular disease of interest. Additional statistical alerts generated by analysis could be evaluated because part of the objective of the IDSAS is to establish the baseline caseload in areas under surveillance.

### Assessment of System Implementation

#### Technical Considerations

Technical barriers were a major challenge during implementation of the IDSAS (Table 3). The system introduced new data collection requirements for field veterinarians. Use of cell phones for data collection required training and ongoing technical support.

**Table 3:** Lessons learned in planning and implementing surveillance systems, Sri Lanka, January 1–September 30, 2009*

Consideration for surveillance in lower-resource settings	IDSAS experience	Generalized lessons
Technical	Cell phones permitted timely collection and transmission of data to the surveillance system. Touch screen interfaces were new technology for field veterinarians.	Use of familiar technologies such as basic cell phones will minimize training time. Cell phones enable timely data collection and transmission.
	Ongoing training was essential. A local research assistant made training more effective, in particular because field veterinarians could learn the system in their native languages.	Developing local expertise at the project outset is invaluable for ensuring sustained technical and logistical support.
Financial	Hardware required for data collection was relatively inexpensive but much more expensive than hardware available in Sri Lanka. Importing cell phones for the project was challenging.	Where possible, hardware that is locally available should be used.
	Open-source software was used when possible, eliminating licensing as a recurring cost but requiring more training and technical skills to maintain.	Open-source software options should be selected over proprietary options to reduce costs and generate technologic capacity.
Political	External funding covered the initial hardware and software costs.	Obtaining external financial support to cover the initial investment required will make implementation more feasible.
	Support at the provincial level was critical for engagement of field veterinarians.	Garnering support at all levels of government is critical at the early implementation phase.
	Engagement of key political stakeholders was essential to alleviate fears about potential for harm caused by novel types of surveillance data.	Early in the design process it is important to discern what the outputs of the system will be and their added value.
Ethical, societal, and cultural	Government officials were initially concerned about data security.	Build appropriate data security into all components of the system.
	It was late in the implementation phase when government stakeholders recognized the potential for additional data uses.	Examples of additional uses of data obtained will generate support for new surveillance initiatives.
	At the onset of the project, field veterinarians were skeptical about the usefulness of data generated by the IDSAS. However, over time they envisaged how the outputs could be used in disease surveillance and in improving their daily veterinary duties.	Adoption of novel surveillance methods requires user acceptance and new technical skills. Time and experience will enable this transition to occur.
	Many farms are geographically isolated making access to field veterinarians difficult.	Quality and quantity of data from surveillance systems are affected by the ability of an animal owner to access animal health services.

*IDSAS, Infectious Disease Surveillance and Analysis System.

#### Financial Considerations

The main costs of the system were associated with data collection hardware. Each phone and GPS extension set cost ≈Can$500. This cost may have been reduced if phones were available for purchase locally. Proprietary software options with different hardware requirements were available but rejected because recurring licensing costs could not be sustained and hardware was a 1-time expense. Although data plans are an ongoing cost, the size of files generated by the IDSAS is typically <1 kilobyte. The cost of data transmission per user per month in Sri Lanka is <Can$5. Investments in hardware and human resources for data collection can be quickly recouped because these resources are extendible to many other fields in which the government of Sri Lanka is involved (e.g., human epidemiology, environmental assessment, disaster planning).

#### Political Considerations

Political support has been the most important factor in the successful implementation and operation of the IDSAS. Animal health reporting standards set by the World Animal Health Organization require member countries to report on a group of animal diseases. The introduction of a new surveillance system as part of a research project resulted in initial confusion about how such a system could fit within existing surveillance networks. A major challenge in the implementation of the IDSAS was drawing the distinction between the IDSAS as a research project and the national animal disease reporting system of the DAPH. Negotiating this challenge was possible with support from key figures in the government and the University of Peradeniya.

#### Ethical, Societal, and Cultural Considerations

During the design and early implementation of the IDSAS, concerns around privacy and data security were addressed promptly as they arose. No information pertaining to animal owners was collected. No personal identifiers from field veterinarians were linked to survey submissions.

## Discussion

The IDSAS was developed on the premise that monitoring animal health can provide information for early warning of emerging infectious diseases and changing disease patterns. Our preliminary results demonstrate enhancement of existing technologic infrastructure. Equipping field veterinarians with the necessary means of communication enabled timely submission of cases, and the skills to make use of these tools helped to build further capacity in animal health surveillance. Weekly reports document increased knowledge and information flow between animal health stakeholders (groups and persons [field veterinarians, administrators, and researchers] whose decision-making and actions are affected by emerging infectious diseases and animal health) in Sri Lanka. Finally, through the IDSAS, major progress was made toward establishing baseline patterns of suspected diagnoses and syndromes in cattle, buffaloes, and chickens.

Uptake of the IDSAS over its initial 9 months of operation resulted in data generation for ≈4,000 interactions between field veterinarians and reports on the animal population. Increasing use of the IDSAS over time was also illustrated by a positive linear trend in submissions. Statistical surveillance of the number of surveys submitted by field veterinarians showed that an upward shift in submissions occurred at approximately week 30. The overall trend was likely caused by field veterinarians acquiring competency with the technology. The shift was likely caused by a combination of reduced number of submissions in weeks 14–16 related to training and examinations, the final stages of the civil war in weeks 19–21, and retraining in week 23. The alarms signaled by the CUSUM analysis illustrate the need for modeling the expected value when surveillance statistics are used.

The distribution of cases highlights 1 of the challenges with this type and many types of surveillance data, which is how to interpret variability in cases in the absence of data on the population at risk. The high number of cattle cases in Nuwara Eliya was expected given prior knowledge of the large number of milk-producing cattle in that region. However, the distribution of cases would be expected to reflect only the true disease incidence in the population if the likelihood of a veterinarian seeing a case in a given species was proportional to the underlying disease distribution in the 3 species in each area. For example, in Nuwara Eliya, cattle raisers might be more inclined to call their veterinarian in the event of a sick cow than a sick chicken. The solution to this problem, if the aim is to establish a predictive, prospective disease surveillance system, is establishing standard patterns of case submission for the population. For this solution to be realized, this system (and others) must be maintained over an extended period within the same geographic areas.

One of the barriers to implementation of the IDSAS in its current form is the cost of hardware and the need for a server administrator. However, since the pilot project was originated in Sri Lanka, a new version of EpiSurveyor has been released. Several major changes have been made: the software now runs on a wide range of standard mobile phones, data can be uploaded to servers administered (by datadyne.org) and analyzed on cell phones, and GPS data can be obtained in EpiSurveyor. These changes drastically reduce the costs of implementing mobile surveillance; the cost per mobile phone unit is reduced substantially, and governments do not need to purchase and administer their own database.

At this time, the DAPH has decided to incorporate the IDSAS into its ongoing disease surveillance efforts, and the system is being run on 2 parallel servers, 1 at the DAPH and the original server that hosts the IDSAS. After this transition period, the system will continue to run only on the DAPH server and may be modified to suit additional surveillance priorities (e.g., goats, swine). The DAPH will not be providing incentives to field veterinarians for participation. Solicitation of further review by field veterinarians (once the system has been transitioned) and monitoring of submissions long term would be valuable.

Beyond data obtained by the IDSAS, this research demonstrates that through developing social capital and technologic capacity, novel surveillance methods can be implemented that are feasible and acceptable in lower-resource settings. These considerations are supplemented with lessons for planning and implementation of surveillance systems. It is hoped that by disseminating the results of this initiative, other governments will tailor the IDSAS to their particular animal health surveillance needs. The collaboration and relationships established in this project should yield further benefits through technical training and pooling of human and physical resources for sustaining and promoting veterinary public health in Sri Lanka. Additionally, the advantages of electronic health surveillance that uses mobile data collection afforded by the IDSAS are immediately known to administrative persons who can affect change in other areas of animal and human health policy and planning.

Developing surveillance capacity in Sri Lanka has generated valuable human resources and relationships that, when coupled with technology, may be the key to early detection of emerging infectious diseases. Field veterinarians are developing a valuable technologic skill set for remote data collection. The data obtained from the IDSAS offers DAPH stakeholders and field veterinarians a new perspective on disease within the animal population and creates new opportunities for dialogue and mutual understanding. Increased communication through training, surveillance reporting, and regular meetings has been a major aspect of improving veterinary public health awareness and is a key result of the IDSAS project. Social capital, although difficult to measure, is a major precursor to successful surveillance in the developing world (*19*). Building social capital under any circumstances takes considerable time. Future development of similar surveillance programs should take this temporal component of project development into consideration to help ensure that new initiatives gain momentum over time. Field veterinarians have indispensable local knowledge about animals in their division. Leveraging this awareness by regular electronic surveillance is a first step toward formalizing this knowledge store to improve surveillance of emerging infectious diseases in Sri Lanka.
